# Melanoma: BRAFi Rechallenge

**DOI:** 10.3390/medicina59050975

**Published:** 2023-05-18

**Authors:** Christoforos S. Kosmidis, Konstantina Papadopoulou, Chrysi Maria Mystakidou, Evanthia Papadopoulou, Stylianos Mantalovas, Nikolaos Varsamis, Charilaos Koulouris, Vasiliki Theodorou, Konstantinos Papadopoulos, Christina Sevva, Petrina Miltiadous, Savvas Petanidis, Eleni Georgakoudi, Eleni Papadopoulou, Sofia Baka

**Affiliations:** 1 European Interbalkan Medical Center, 10 Asklipiou Street, 55535 Pylaia, Greece; dr.ckosmidis@gmail.com (C.S.K.);; 2 3rd Surgical Department, “AHEPA” University Hospital of Thessaloniki, School of Medicine, Aristotle University of Thessaloniki, 1st St. Kiriakidi Street, 54621 Thessaloniki, Greece; 3 1st Department of Internal Medicine, G. Papanikolaou General Hospital of Thessaloniki, 57010 Thessaloniki, Greece; 4 Medical School, Faculty of Health Sciences, Aristotle University of Thessaloniki, 54124 Thessaloniki, Greece; 5 Shakolas Educational Centre for Clinical Medicine, University of Cyprus, Old Road Nicosia-Lemesos 215/6, 2029 Nicosia, Cyprus; 6 Laboratory of Medical Biology and Genetics, Department of Medicine, Aristotle University of Thessaloniki, 54124 Thessaloniki, Greece

**Keywords:** targeted therapy, BRAFi rechallenge, metastatic melanoma, BRAF V600 mutation, BRAF inhibitor

## Abstract

Melanoma is the most aggressive type of skin cancer. Half of melanoma cases are characterized by the mutation BRAF V600. The case presented concerns a 41-year-old patient with locally advanced melanoma, being positive in mutation BRAF V600. The patient underwent surgery and received additional targeted therapy as part of a clinical study. In subsequent disease progression, immunotherapy was used. When the disease progressed again while the patient was in a good performance status, targeted therapy was administered again, and a good response was noted, making the patient reach a statistically significant overall survival, exceeding four years. Targeted therapy has proven to be an important tool in the treatment of melanoma. The use of BRAFi targeted therapy does not exclude the option of readministration at subsequent disease progression (BRAFi rechallenge). Preclinical models suggest that the resistance mechanism of cancer cells to BRAFi therapy bends, as these cell clones lose their evolutionary advantage after stopping BRAFi. Cell clones sensitive to BRAFi may then outcompete, making the treatment effective again. Therapeutical dilemmas in the management of patients with locally advanced melanoma that progresses to metastatic cancer are discussed.

## 1. Introduction

Melanoma is a very aggressive type of skin cancer. Over the past years, its incidence has increased steadily. Despite progress in therapeutic approaches, the overall survival rates have remained relatively low. The progress in the quest for better management is obvious; novel treatment options have developed and gained approval, including targeted therapy and immunotherapy.

Targeted therapy has been a major breakthrough in the last decade, addressing melanoma BRAFV600-mutant tumors. The COMBI-AD clinical trial advanced the treatment of BRAFV600E/K-mutant melanoma. In this trial, patients with Stage III melanoma, who were positive for the BRAFV600E/K mutation, received oral dabrafenib plus trametinib after the surgical resection as adjuvant therapy. The results of five-year follow up are posted, showing obvious benefit in relapse-free survival (52% vs. 36%) and distant-metastasis-free survival (65% vs. 54%) [1]. Despite the beneficial outcome that has been described, many patients receiving targeted therapy, as well as immunotherapy, experience resistance quickly, raising concerns about the next treatment step. There is intense research to forestall resistance, leading to some conclusions in clinical practice, such as the BRAFi-MEKi rechallenge [2,3,4].

This case report describes a patient who was diagnosed with stage IIIC melanoma. Based on the staging, it was considered a high-risk type of disease, with a five-year survival rate less than 50%. This patient was treated with targeted therapy, immunotherapy, and then targeted therapy rechallenge, reaching five-year survival mostly with a good quality of life (based on PFS [Performance Status Scale]). Rechallenge with targeted therapy is highlighted hereby; it offered survival benefit when treatment options had been eliminated, while the patient remained in good performance status.

## 2. Case Presentation

A 41-year-old man presented to our clinical department in February 2014 with a rapidly changing black mole on his left leg. Initially, it was a flat brownish macule, which he had for many years. The patient reported that the lesion had become slightly raised. Physical examination and review of systems showed nothing remarkable, and there was no lymphadenopathy. When asked about past medical history or family history, he did not report any relevant issues. 

The excision biopsy was performed, and the histopathologic examination demonstrated obvious proliferation of atypical melanocytes in all the levels of the epidermis (pagetoid spread). Additionally, the patient demonstrated atypical melanocytes, both individually and in small nests extended into the papillary dermis. The histologic diagnosis was consistent with malignant melanoma of the superficial spreading type, and the margins were clear (0.3 cm). The Breslow thickness was 4.3 mm (Clark level IV). There was no vascular invasion or satellite lesions. Appropriate histochemistry and immunohistochemistry techniques were used to confirm the diagnosis: HMB45(+), S-100 (+). In March 2014, a wider excision was performed, and the sentinel lymph node biopsy was positive for disease. Lymph nodes clearance did not show further lymph nodes to be positive. Finally, referring to the BRAF mutation, the patient was positive for the mutation–V600E.

With the above data, in April 2014, the patient took part in the COMBI-AD clinical study. This study concerns the evaluation of adjuvant treatment with BRAF and MEK inhibitors–Dabrafenib and Trametinib, and it provided this treatment for high-risk BRAF V600 mutation-positive melanoma after surgical resection. The patient was introduced to the treatment, with no adverse events. The clinical trial has already posted some results, referring to the relapse-free survival and the distance metastasis-free survival [1].

**Figure 1:**
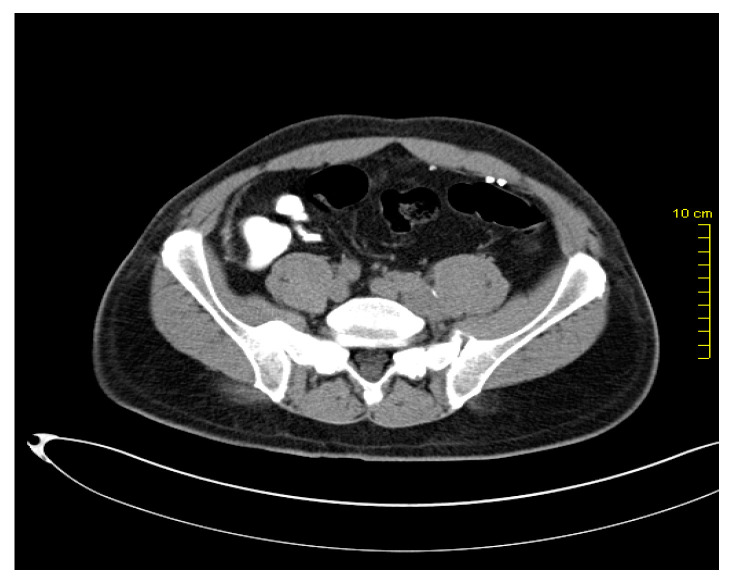
Pelvic lymph node metastases.

**Figure 2:**
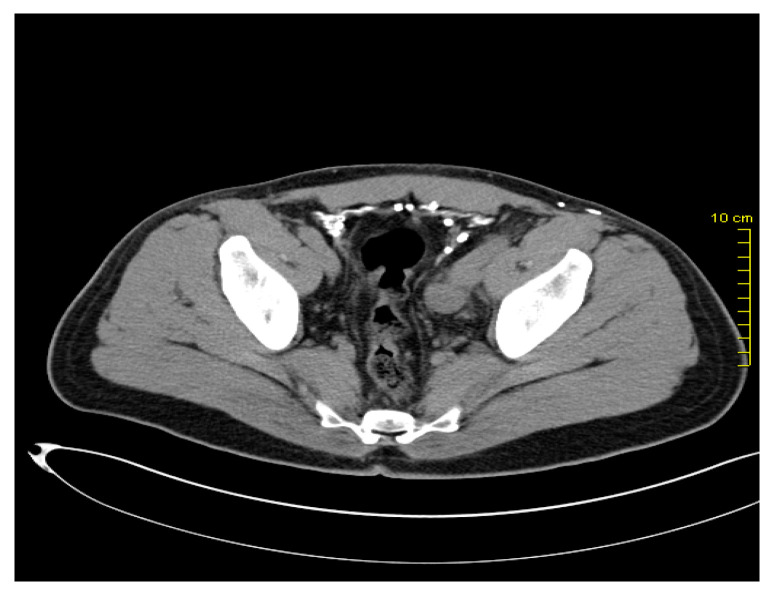
Pelvic lymph node metastases.

After two years, in March 2016, during the re-examination of the patient, pelvic lymphadenopathy was detected (Figure 1 and Figure 2), which, with the subsequent biopsy, was identified as disease progression with Vimentin(+), Melan C(+), S-100 (+), and AE1/AE3(-).

**Figure 3:**
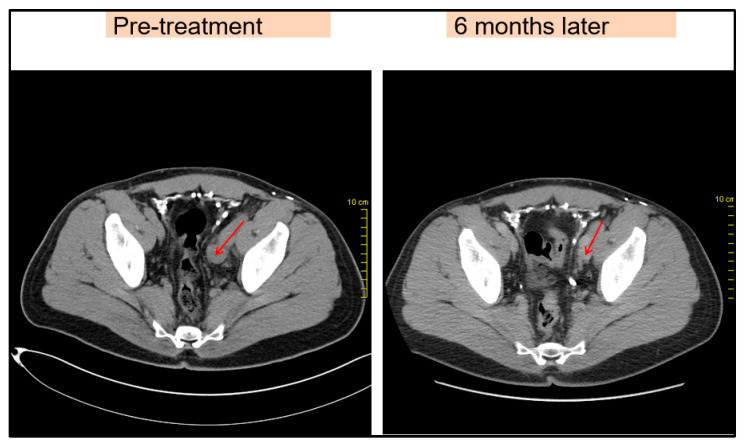
Pelvic lymph node metastases before and after immunotherapy, comparison showing regression (red arrow).

Due to disease progression, in May 2016, the patient started receiving an immunotherapy regimen as follows: Nivolumab 1 mg/kg and Ipilimumab 3 mg/kg every three weeks for four cycles, as well as maintenance immunotherapy with Nivolumab 3 mg/kg every two weeks. The patient responded to this new regimen with a significant regression of the lymph node metastasis, visible on imaging (Figure 3). During immunotherapy, the patient experienced adverse reactions. He presented grade I-II lethargy, grade I myalgia, and grade I-II diarrhea during Nivolumab + Ipilimumab combination therapy. Subsequently, with maintenance monotherapy, the patient developed grade I lethargy, grade I-II skin rash, grade I toxic ophthalmopathy, and hypothyroidism. These toxic effects were treated appropriately. Eye drops and topical corticosteroids were given for ophthalmopathy. Concurrently, the patient was treated with Levothyroxine, 50–75 μg/day. The patient remained in good condition with an excellent performance status (Karnofsky PS = 0) and continued to work during treatment.

One year after starting immunotherapy, in May 2017, the patient experienced progression of the disease. Specifically, the CT scans showed nodular metastatic sites in the lungs, bilaterally, and metastasis in the liver. At the same time, the pelvic lymph nodes showed deterioration.

At this point, targeted therapy—BRAFi and MEKi—as well as immunotherapy, had already been used. Metastatic melanoma does not respond well to chemotherapy, as patients do not seem to benefit much in terms of overall survival. Therefore, having used the “powerful weapons” in the fight against melanoma, the management of this recurrence has raised concerns. Targeted therapy, which the patient had started years ago, was considered a good choice, as the tumor cell resistance mechanisms that may have formed may have been eliminated by stopping BRAFi and MEKi for a significant period of time [2,3,5,6]. 

The patient, therefore, restarted the regimen of targeted therapy with BRAF and MEK inhibitors (BRAFi and MEKi retreatment), specifically with Vemurafenib and Cobimetinib, from June 2017. The results showed a significant improvement, being that, in October 2017, the CT scan showed complete disappearance of the lung metastases, and the disease remained stable, since then, for several months. Regarding treatment toxicity, the patient developed a grade II skin rash and fever, which were treated with dose modification.

**Figure 4:**
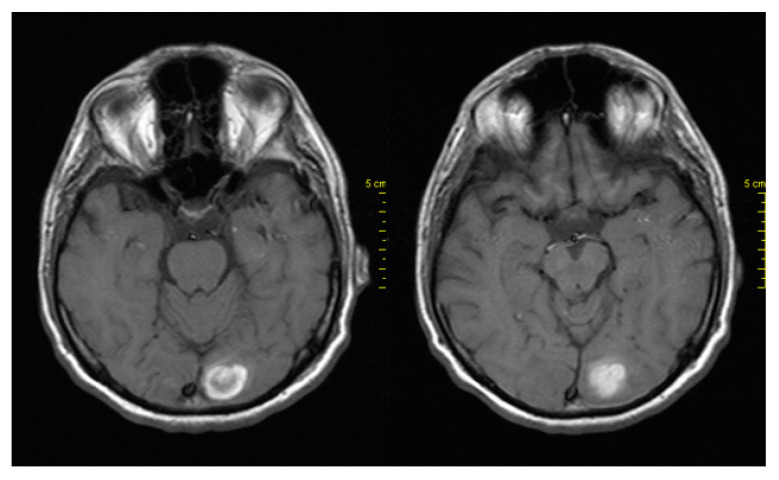
MRI showing brain metastases in the left occipital lobe.

In April 2018, due to headache and dizziness, the patient underwent a neurological examination and brain imaging. Brain CT and MRI showed new brain metastases in the left occipital lobe (Figure 4). The next step was brain radiotherapy in May 2018, with 3000cGy to the whole brain and 600cGy boost. The patient subsequently continued receiving BRAFi + MEKi and showed clinical improvement. A CT scan of the brain performed after two months showed stability of the disease.

The patient has since remained in good performance status—PS:0—for five months. In November 2018, due to clinical worsening of the disease, he stopped targeted therapy and continued with symptomatic treatment with corticosteroids. The overall patient survival reached five years from the initial disease onset, with good quality-of-life—Karnofsky PS:0—for a long time. The prospect of administering targeted therapy a second time to the same patient (BRAFi rechallenge/retreatment) is recommended, with optimistic results in the mentioned patient, and also in relevant preclinical and clinical studies that have been taking place in recent years.

## 3. Discussion

Melanoma is a type of skin cancer, which—while its incidence is constantly increasing—is better treated with the evolution in oncology therapy. The overall survival of these patients increased statistically by the emergence of immunotherapy in melanoma and the development of targeted therapies [1]. Patients with BRAF-mutated melanoma are being treated more and more effectively by employing targeted therapy and immunotherapy. Specifically, for BRAFV600-mutant patients, the development of BRAF and MEK inhibitors was highly encouraging. These advances were made in the last decade, and the patient described above derived benefit from COMBI-AD, a clinical trial that brought dabrafenib and trametinib into the adjuvant therapy setting [1]. Still, nearly all patients treated with BRAFi and MEKi eventually develop resistance. Thus, studying the results of targeted therapy rechallenge seems crucial, based on the absence of further efficient treatment regimens, other than immunotherapy. Combination treatments are also studied currently as an alternative when resistance occurs [4].

The path to precision-oriented medicine clearly includes the molecular targeting offered by BRAFi-targeted therapy, but also the recognition of the patient’s prognostic profile and the time point at which each treatment regimen can act. Consequently, the decision to re-administer the aforementioned targeted therapy should be made for a selected group of patients in a selected timeframe. Studies show that prognostic factors for patients treated with combination BRAF and MEK inhibition include PS, tumor burden (number of metastatic sites), and LDH level [3]. Specifically, patients with less than three metastatic sites, low LDH, and a good score on PS scales, were the ones who seemed to benefit from a BRAFi–MEKi rechallenge–resulting in a better overall survival.

It was stated that interruption of BRAFi therapy for longer than 12 weeks—whether immunotherapy-mediated or not—appears to be able to confer an advantage to its re-administration [7,8]. The minimum time length of the break is still not well established. In a retrospective study, it has been documented that the duration of the interval between BRAFi cessation and rechallenge is not associated with survival, suggesting that a specifically long drug holiday is not required for resensitisation [3]. Further comparative studies will hopefully clarify the therapeutical outcome regarding the interval duration. 

At a molecular level, many resistance mechanisms are reported to reactivate the MAPK pathway, offering a growth advantage while on inhibitor drugs. Some well established mechanisms include BRAFV600E amplification, different splicing of BRAFV600E, and other mutations in the BRAF pathway [9,10]. Adaptive resistance also develops in the absence of genomic alterations involving transcriptional changes by epigenetic mechanisms that trigger the epithelial-to-mesenchymal transition (EMT), melanocyte dedifferentiation, and neural crest stem cell-like reemergence [11,12]. Additionally, activation of autophagy is one of the known mechanisms of resistance to BRAF/MEK inhibitors.

Based on the above resistance mechanisms, studies have been made in the quest for a way to escape resistance. Hydroxychloroquine is an autophagy inhibitor, and it has been suggested in preclinical melanoma models, where it could decrease resistance to BRAF/MEK inhibitors. It has been evaluated in vivo and in safety studies, but clinical conclusions have not been noted yet [13]. Another way of escaping resistance that has been proposed is “Intermittent Treatment” with BRAF and MEK inhibitors. This is a method that has been suggested because of the phenotypic plasticity in drug resistance, but it has not been proven to show benefit. Two recent phase II trials revealed worse progression-free survival in patients with melanoma, who were treated intermittently with BRAFi and MEKi, compared with those treated with continuous therapy, with no difference in overall survival [12]. In conclusion, the considerable benefit of targeted therapy rechallenge in melanoma has triggered different approaches in the BRAFi and MEKi methods of administration in the quest for the most effective treatment in terms of delaying resistance. Still, no clinically proven alternative method for escaping resistance has been developed yet. Thus, when we are faced with progression after targeted therapy and checkpoint inhibitors, the best next step seems likely to be targeted therapy rechallenge, with that being in the context of a patient with good PFS and less than three metastatic sites.

BRAFi rechallenge in advanced-stage melanoma is supported both by cases, such as the aforementioned, and by preclinical and clinical studies looking for the mechanisms of resistance and resensitization of cancer cells to targeted therapies [5,14]. The clues are clear; more than 30% of patients who progress to BRAF or MEK inhibitors have been reported to show a second clinical response after a drug holiday period [3,7]. It is a new plausible treatment option for selected patients with BRAF-mutated melanoma who had, at first, responded to BRAFi, who have subsequently progressed, and who have completed a following treatment. The best clinical response is noted by Valpione et al. when administering combination therapy in patients with less than three metastasis sites [3]. It is supported because of the promising survival data, the known toxicity profile, and the absence of favorable treatment alternatives. The recognition of different prognostic groups offers methods for the design of clinical trials assessing intermittent dosing approaches or rechallenge therapy with BRAFi after a different regimen.

## 4. Conclusions

Treatment of melanoma has significantly advanced in the light of new therapies: immunotherapy and targeted therapy. However, many patients experience early resistance and progression. Patients, such as the one we described, who are young and keep a good PFS, are eligible for further treatment. Still, effective treatment options other than the aforementioned remain few in number. This case report describes the management of a patient who benefited from adjuvant targeted therapy, immunotherapy, and, when he had progressed, he was administered targeted therapy again (rechallenge), reaching a relatively long OS and a good quality-of-life for most of time. In the context of progression to both targeted therapy and checkpoint inhibitors, patients who have a good PFS, low tumor burden (less than three metastatic sites), and relatively low LDH, are noted to have the most favorable outcome with this treatment approach of rechallenge with combination BRAFi and MEKi [2,3]. Further research is still expected to shed light on this treatment option regarding best timepoint of readministration, possible combination treatment, and more detailed patient selection for targeted therapy rechallenge.

## Data Availability

No new data were created or analyzed in this study. Data sharing is not applicable to this article.
